# The Present and the Future of Medical Therapies for Adenomyosis: A Narrative Review

**DOI:** 10.3390/jcm12196130

**Published:** 2023-09-22

**Authors:** Gaby Moawad, Youssef Youssef, Arrigo Fruscalzo, Hani Faysal, Mira Kheil, Paul Pirtea, Benedetta Guani, Jean Marc Ayoubi, Anis Feki

**Affiliations:** 1 Department of Obstetrics and Gynecology, George Washington University, Washington, DC 20037, USA; 2 The Center for Endometriosis and Advanced Pelvic Surgery, Washington, DC 22101, USA; 3 Division of Minimally Invasive Gynecology, Department of Obstetrics and Gynecology, Maimonides Medical Center, Brooklyn, NY 11220, USA; 4 Department of Obstetrics and Gynecology, HFR—Fribourg, Chemin des Pensionnats 2-6, 1708 Fribourg, Switzerland; arrigo.fruscalzo@h-fr.ch (A.F.);; 5 Department of Obstetrics and Gynecology, Indiana University, Indianapolis, IN 46202, USA; 6 Department of Obstetrics and Gynecology, Henry Ford Health, Detroit, MI 48202, USA; 7 Department of Obstetrics and Gynecology and Reproductive Medicine, Hopital Foch–Faculté de Médecine Paris, 92150 Suresnes, France

**Keywords:** adenomyosis, medical treatments, progesterone, intrauterine devices, gonadotropin releasing hormones

## Abstract

Uterine Adenomyosis is a benign condition characterized by the presence of endometrium-like epithelial and stromal tissue in the myometrium. Several medical treatments have been proposed, but still, no guidelines directing the management of adenomyosis are available. While a hysterectomy is typically regarded as the definitive treatment for adenomyosis, the scarcity of high-quality data leaves patients desiring fertility with limited conservative options. Based on the available data, the levonorgestrel-IUD appears to offer the most favorable outcomes. Other treatments, including GnRH antagonists, dienogest, prolactin, and oxytocin modulators, show promise; however, further data are required to establish their efficacy definitively. Furthermore, there are many emerging therapies that have been developed that seem worthy of consideration in the near future. The aim of this narrative review was to explore the current medical treatments available for adenomyosis and to provide a glimpse of future therapies under assessment. For this scope, we performed a literature search on PubMed and Medline from incept to September 2022 using the keywords: “medical treatment”, “non-steroidal anti-inflammatory”, “progesterone intrauterine device”, “dienogest”, “combined oral contraceptives”, “gonadotropin releasing hormone agonist”, “gonadotropin releasing hormone antagonist”, “danazol”, “aromatase inhibitors”, “ulipristal acetate”, “anti-platelet therapy”, “dopamine”, “oxytocin antagonists”, “STAT3”, “KRAS”, “MAPK”, “micro-RNA”, “mifepristone”, “valproic acid”, “levo-tetrahydropalamatine”, and “andrographolide”. The search was limited to articles in English, with subsequent screening of abstracts. Abstracts were screened to select relevant studies.

## 1. Introduction

Uterine Adenomyosis is a benign condition histologically defined as the presence of endometrium-like epithelial and stromal tissue in the myometrium, along with enlargement of the uterus [1,2]. Its prevalence is estimated to be around 1%, with an incidence of 29 per 10,000 person–years [3]. Like endometriosis and leiomyoma, adenomyosis is most identified in women aged 41–45 years and is more prominent in the African American population [1,2,3]. The exact etiology of adenomyosis remains unclear, despite multiple proposed theories, such as Müllerian rests, metaplasia of stem cells, genetic mutations, and endometrial invagination into the myometrium [4,5,6]. New theories related to endometriosis pathophysiology could change our current understanding of adenomyosis, as adenomyosis and endometriosis are closely linked, both being estrogen-dependent and often found concomitantly in patients [7,8,9]. One of these newer theories is that genetic-epigenetic changes affect intracellular aromatase activity causing intracellular estrogen production with the subsequent development of inflammatory, fibrotic endometrial-like tissue outside the uterus [10]. It is important to note that although adenomyosis and endometriosis share similar histological features and molecular changes, they differ in pathogenesis, location, and clinical features [11,12].

It is worthy of note that next-generation sequencing technologies have boosted research for new markers for signaling pathways involved in the pathogenesis of adenomyosis. For example, there is increasing evidence concerning the role of KRAS mutations in the pathogenesis of adenomyosis [13]. Interestingly, such mutation profiles found in adenomyosis seem to be shared with endometriosis, supporting the hypothesis of a similar origin of these two pathologies [14]. These mutations seem to be involved in progesterone resistance impacting cell proliferation, survival, differentiation, and migration abilities. Furthermore, they seem to favor the activation of further molecular pathways, involved in inflammation, neurogenesis, fibrosis, and neovascularization, some of the most important processes underpinning the development of these pathologies [15]. 

Adenomyosis presents clinically with debilitating symptoms such as menorrhagia, chronic pelvic pain, dysmenorrhea, and infertility, requiring treatment [16]. Diagnosis of adenomyosis is made via transvaginal ultrasonography (US) or MRI but definitive diagnosis requires histopathological evidence. US findings include heterogeneous myometrium, myometrial cysts, and asymmetric myometrial thickness, in addition to sub-endometrial echogenic linear striations [17,18,19,20,21]. MRI findings include high-intensity foci representing hemorrhage and increased thickness of the junctional zone representing smooth muscle hyperplasia with accompanying heterotopic endometrial tissue [22]. Studies comparing the effectiveness of transvaginal US and MRI have demonstrated the latter to be equal, if not superior, in the diagnosis of adenomyosis [23,24,25,26,27].

Currently, no guidelines directing the management of adenomyosis are available, despite numerous treatments, both medical and surgical in nature [28,29]. Although hysterectomy provides a definitive cure, it is not the method of choice for patients willing to preserve future fertility or those who are not medically fit for surgery [30]. In light of the increasing trend of late childbearing, various pharmacological therapies and fertility-preserving surgeries have emerged.

Studies have found that sex hormone anomalies including local hyper-estrogenism due to the activation of aromatase and sulfatase, decreased progesterone receptors, progesterone resistance, inflammation, altered cell proliferation, and neuro-angiogenesis are likely key pathogenic mechanisms of pain, abnormal uterine bleeding (AUB), and infertility in adenomyosis [1,31]. Thus, controlling the hormonal medium could be the basis for adenomyotic lesion regression [32].

The aim of this review is to explore the current medical treatments available for adenomyosis in addition to future, prospective therapies.

## 2. Materials and Methods

**Figure 1:**
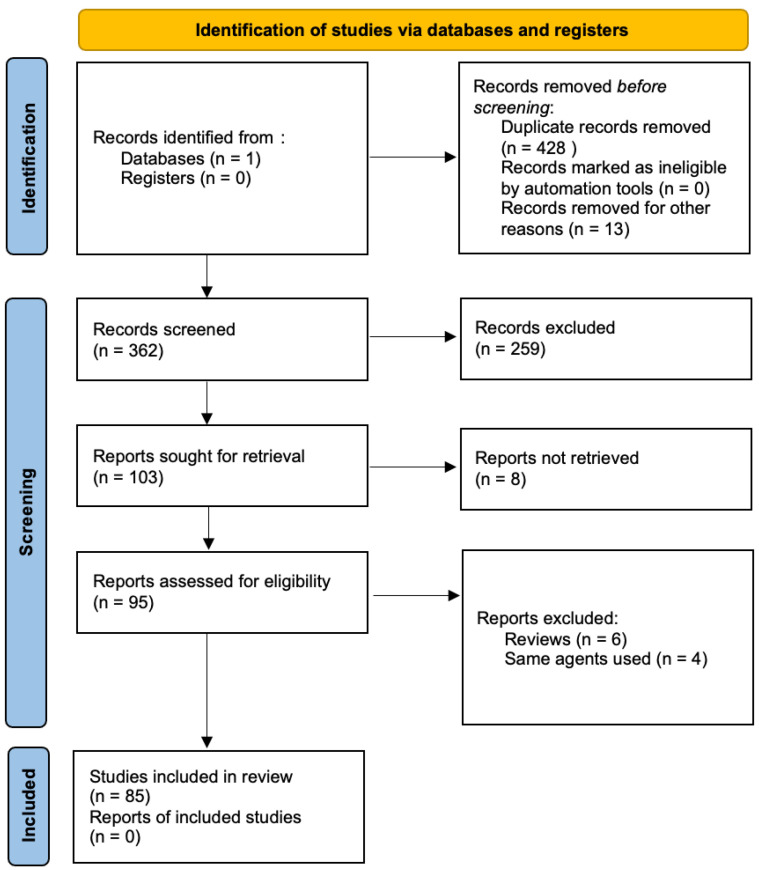
Flow diagram of study identification and selection.

This article is a narrative review. To conduct our review, we searched PubMed with terms including “adenomyosis” and “treatment” to identify the multiple treatment modalities involved in adenomyosis. After identifying the different medical treatment, more in-depth search strategies were set up for the different therapies, combining “adenomyosis” with the following terms: “medical treatment”, “non-steroidal anti-inflammatory”, “progesterone intrauterine device”, “dienogest”, “combined oral contraceptives”, “gonadotropin releasing hormone agonist”, “gonadotropin releasing hormone antagonist”, “danazol”, “aromatase inhibitors”, “ulipristal acetate”, “anti-platelet therapy”, “dopamine”, “oxytocin antagonists”, “STAT3”, “KRAS”, “MAPK”, “micro-RNA”, “mifepristone”, “valproic acid”, “levo-tetrahydropalamatine”, and “andrographolide”. The search was limited to articles in English, with subsequent screening of abstracts. Abstracts were screened to select relevant studies. Inclusion criteria were randomized controlled trials, case controls, cohorts, case series, case reports, and systematic reviews and meta-analyses. Exclusion criteria were any language other than English, letters to editors, and video articles. We included 85 studies for this purpose (Figure 1).

## 3. Results

### 3.1. Classical Treatments

#### 3.1.1. Non-Steroidal Anti-Inflammatory Drugs (NSAIDs)

NSAIDs have been proven to be effective in treating dysmenorrhea, despite insufficient evidence to determine the safest and most effective agent in that class [33]. A Cochrane database systematic review demonstrated a statistically relevant decrease in heavy menstrual bleeding when comparing NSAIDs to placebo, despite being less effective than hormonal treatments or tranexamic acid [34]. Nevertheless, NSAIDs and other analgesics remain the sole treatment option in women with adenomyosis interested in pregnancy [35]. In symptomatic women with no interest in conceiving, hormonal treatments are preferred to address chronic abnormal uterine bleeding and pain, with NSAIDS prescribed only in acute exacerbations [35].

#### 3.1.2. Levonorgestrel-Releasing Intra-Uterine Device (LNG-IUD)

LNG-IUDs diffusing 20 µg/day of levonorgestrel are often used in women with adenomyosis experiencing abnormal uterine bleeding, despite being originally designed for long-term contraception [35,36]. They exert their control via decidualization and atrophy of the endometrial tissue by creating a hypoestrogenic state and by downregulating estrogen receptors due to high progestin release [37,38]. Ozdegirmenci et al. found LNG-IUD to be comparable to hysterectomy after measuring hemoglobin levels at 6 months and 1 year of treatment [39]. Another study noted that LNG-IUDs were superior to combined oral contraceptive pills in pain reduction and uterine volume reduction in patients with adenomyosis [40]. In a 3-year follow-up study, Sheng et al. demonstrated that LNG-IUD reduced menstrual bleeding, pain, and uterine volume, in addition to achieving an overall satisfaction rate of 72% [41].

LNG-IUDs are currently the best-evaluated and most efficacious treatment of adenomyosis-related symptoms with a high rate of symptom improvement, minimal sideeffects, and an improvement in the quality of life that is similar to that of a hysterectomy [35].

#### 3.1.3. Progestins

Progestins exert an anti-proliferative and anti-inflammatory effect, leading to the decidualization and atrophy of endometrial tissue, with a subsequent significant reduction in bleeding [28].

Dienogest (DNG) is a synthetic progestogen with properties of 19-norprogesterone and 17α-hydroxyprogesterone derivatives used in the long-term treatment of endometriosis [35]. It has been proven to improve primary and secondary dysmenorrhea [42]. Despite being used for the treatment of adenomyosis-related symptoms, there is currently no proper therapeutic management protocol for its use in the treatment of adenomyosis [28]. In a recent randomized, double-blind placebo-controlled trial, DNG demonstrated a significant decrease in patient pain scores, with subsequent improvement in quality of life, pain levels, and good tolerability as a long-term treatment [43,44].

Danazol is a synthetic modified progestogen that reversibly inhibits the synthesis of LH and FSH and has a weak androgenic effect [45]. It is used in endometriosis to shrink ectopic endometrial tissue in addition to reducing aromatase expression in the eutopic endometrium [46]. A recent study by Igarashi et al. found that Danazol-loaded IUDs improved dysmenorrhea and decreased myometrial thickness with fewer side effects in patients with adenomyosis when compared to oral danazol [47,48,49]. An improvement in dysmenorrhea and fertility was also appreciated in patients treated with a danazol vaginal ring [50].

#### 3.1.4. Gonadotropin Releasing Hormone (GnRH) Agonist and Oral Contraceptive Pills (OCP)

GnRH is a decapeptide secreted by the hypothalamic neurons that acts on receptors in the anterior pituitary gland. Continuous prolonged stimulation by GnRH agonists leads to a central downregulation with the suppression of gonadotropin secretion, ultimately leading to the induction of a hypoestrogenic state in addition to an antiproliferative effect within the myometrium [51]. Additionally, combined oral contraceptive pills were found to be effective in treating dysmenorrhea [52]. Both OCPs and GnRH agonists are used as suppressive hormonal therapies to induce the regression of adenomyosis and improve the severity of symptoms [53].

Interestingly, Mansour et al. demonstrated the regression of adenomyotic lesions on imaging after treating one patient with a course of OCPs. Moreover, they noted the resolution of adenomyotic lesions and chronic pelvic pain in three patients treated with leuprolide acetate, a GnRH agonist [54]. A randomized non-blind clinical trial demonstrated that both GnRH agonists and Letrozole led to uterine and adenomyoma volume reduction in addition to a decrease in chronic pelvic pain [55].

GnRH agonists are also indicated to improve the chances of pregnancy in women with adenomyosis [56]. The highest pregnancy rate has been reported in women undergoing frozen embryo transfer after GnRH agonist treatment [57]. It is important to note, however, that the use of GnRH agonists for pain and bleeding should be restricted to short-term only due to possible menopausal effects [58].

#### 3.1.5. GnRH Antagonists

**Figure 2:**
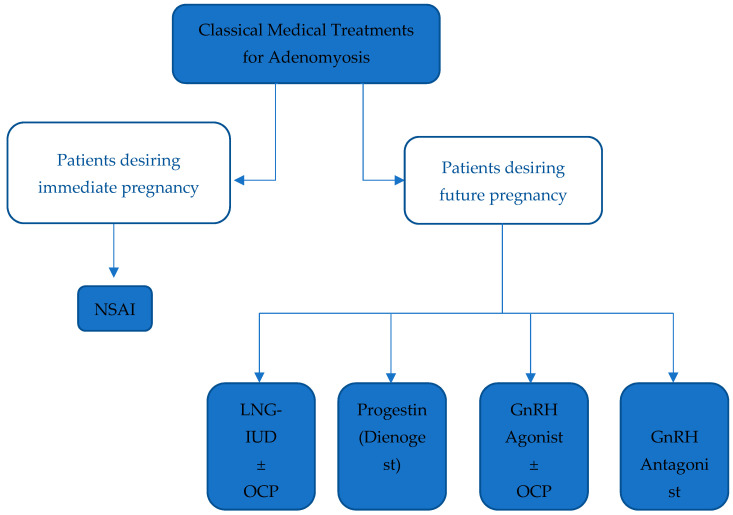
Summarizing classical medical treatments for Adenomyosis. Legend: NSAID: Non-steroidal anti-inflammatory drug, LNG-IUD: Levonorgestrel-releasing intrauterine device, OCP: Oral contraceptive pill, GnRH: Gonadotropin-releasing hormone.

GnRH antagonists are peptide compounds that share a structure like that of natural GnRH and have an immediate antagonist effect on GnRH receptors in the pituitary gland, inhibiting gonadotropin secretion [35]. A case report by Donnez et al. describes a patient who, after failing a course of ulipristal acetate, a Selective Progesterone Receptor Modulator (SPRM), was prescribed Linzagolix, a GnRH antagonist. Treatment with Linzagolix reduced adenomyotic lesion size and dysmenorrhea burden, ultimately leading to improved quality of life [59]. Another GnRH antagonist, Elagolix, is currently being developed for the long-term treatment of endometriosis and uterine leiomyomas [55,60,61,62]. Elagolix has been shown to regress the size of fundal adenomyoma, with an improvement in clinical symptoms and the resolution of pelvic pain [63]. The main advantage of GnRH antagonists over GnRH agonists is their ability to maintain sufficient estradiol levels to avoid bone demineralization and estrogen deprivation symptoms [64] (Figure 2).

### 3.2. Prospective and Future Treatments 

#### 3.2.1. Aromatase Inhibitors

Aromatase P450 is an enzyme that catalyzes the conversion of androgens to estrogens. It is expressed physiologically in the granulosa cells of growing follicles amongst other cells [35]. Aromatase inhibitors that halt the production of estrogen are used in adenomyosis to suppress the hormonal medium favoring disease progression [65]. In their randomized control trial, Badawi et al. found GnRH agonists and aromatase inhibitors to be equally effective in reducing adenomyosis and symptoms burden [55]. Decreases in uterine and adenomyoma volumes were comparable among both treatment arms, suggesting that aromatase inhibitors are as effective as GnRH agonists [55]. When combined, however, both agents further reduced uterine volume by 60% on imaging after 8 weeks of treatment [66].

#### 3.2.2. Selective Progesterone Receptor Modulators (SPRMs)

SPRMs are modified synthetic steroids derived from norethindrone that can interact with progesterone receptors to either activate or repress gene transcription in a tissue-specific manner [35]. SPRMs have been proven to reduce the size of uterine fibroids, stop endometrial bleeding and changes, and suppress the luteinizing hormone peak while maintaining normal follicle-stimulating hormone levels [67,68,69,70,71,72]. The two most commonly prescribed SPRMs are Ulipristal acetate (UPA) and Mifepristone [35].

In a randomized control trial by Capmas et al., UPA was administered to a group of 30 women with adenomyosis who were then compared to a control group of 10 patients [73]. There was a significant decrease in the pictorial blood loss assessment chart in the treatment group, with 95.24% of patients scoring below 75, compared to scoring above 100 before the intervention (*p* < 0.01) [73]. Pain also improved after treatment; however, no significant differences in pain or blood loss were found at a 6-month follow-up [73]. Interestingly, UPA was found to worsen adenomyosis in some cases. Conway et al. described six women misdiagnosed with fibroids who, after treatment with UPA, experienced worsening pain with the enhancement of adenomyotic lesions on ultrasound [74]. These findings were also reported in other studies [75].

Mifepristone is one of the first and most widely used SPRMs. Its affordability and low-risk profile can be of great advantage to patients since adenomyosis requires long-term medical therapy [76]. Wang et al. compared different dosages of mifepristone to a placebo. Their outcome of interest was the immunohistochemical expression of caspase-3 in the eutopic and ectopic endometrium in women with adenomyosis [77]. Their results showed an increase in caspase-3 expression in women treated with mifepristone. This finding suggests that mifepristone could induce eutopic and ectopic endometrial cells to undergo apoptosis by activating caspase-3 expression [77]. Che et al. described decreased levels of Interleukin-6 and Tumor Necrosis Factor-α from endometrial epithelial and stromal cells, restricted infiltration and degranulation of mast cells in eutopic and ectopic endometrium, and a decrease in the density of nerve fibers [78]. They concluded that mifepristone can be used to address dysmenorrhea in adenomyosis patients [78]. Further studies have emerged supporting the use of mifepristone in the treatment of adenomyosis, via the downregulation of immune checkpoint proteins normally upregulated in adenomyosis tissues [79].

#### 3.2.3. Antiplatelet Therapy

Serial immunohistochemistry analyses of ectopic endometrium in mouse models demonstrated that platelet activation coincided with the induction of the TGF-β/Smad signaling pathway in adenomyosis, ultimately leading to fibrosis and smooth muscle metaplasia (Shen et al. unpublished data) [80,81]. These findings were similarly demonstrated in human adenomyosis [82]. Zhu et al. induced adenomyosis in mice to test this theory. They then administered Ozagrel, a thromboxane A2 inhibitor [83], and a rat anti-mouse GPIbα polyclonal IgG antibody and its isotype-matched non-immune rat anti-mouse IgG antibody [81,83]. They found that antiplatelet therapy resulted in suppressed myometrial infiltration, improved generalized hyperalgesia, and reduced uterine contractility in mice with induced adenomyosis [81]. Further studies evaluating the effectiveness of anti-platelet therapy on humans with adenomyosis are needed, despite promising results with mice models.

#### 3.2.4. Dopamine Agonist

Adenomyosis in mice models can be stimulated hormonally by inducing hyperprolactinemia [84]. Prolactin and its receptors are increased in adenomyotic tissue, suggesting an association between prolactin and adenomyosis [85]. Bromocriptine, a dopamine agonist and prolactin inhibitor when administered orally, is recognized for its notable prevalence of adverse effects, leading to a 10% discontinuation rate among patients. Frequently reported side effects encompass gastrointestinal symptoms, headaches, dizziness, sinus congestion, and alterations in orthostatic blood pressure and heart rate. Nonetheless, prior research indicates that the use of vaginal bromocriptine is associated with minimal gastrointestinal symptoms and lacks orthostatic or heart-rate fluctuations. Notably, headaches and dizziness appear consistent irrespective of the administration route, implying that these manifestations may be attributed to the central action of bromocriptine (Kletzky et Vermesh 1989).

Andersson et al. evaluated the effectiveness of bromocriptine, a dopamine agonist and prolactin inhibitor, on women with adenomyosis [74,75]. In their pilot study, patients with adenomyosis who were treated with 9 months of vaginal bromocriptine reported improved pain, self-image, symptom severity, and higher health-related quality of life [86]. In their second study, Andersson et al. evaluated bromocriptine therapy using imaging [87]. After 6 months of treatment, ultrasounds demonstrated a significantly thinner maximal junction zone; however, no significant differences could be appreciated on MRI [87]. Interestingly, asymmetric wall thickening was present in 72% of patients at baseline, and only in 33% after 6 months of treatment in the US [87].

#### 3.2.5. Oxytocin Antagonists

Women experiencing primary dysmenorrhea have been found to have higher oxytocin and vasopressin plasma levels, inducing an increase in myometrial peristalsis via oxytocin receptors (OTR) and vasopressin V_1a_ receptors [35]. Nie et al. demonstrated that the immunoreactivity of OTR was increased in endometrial stromal and epithelial cells in addition to myometrial and vascular cells in ectopic adenomyosis foci, concluding that OTR expression in epithelial cells was correlated with the severity of dysmenorrhea [88]. Atosiban, an OTR antagonist, has been proven to reduce myometrial contractility and pain symptoms in women with primary dysmenorrhea [89]. Epelsiban, another OTR antagonist, was tested on a population of healthy women and was found to be well tolerated [90]. Unfortunately, no further clinical studies have been published concerning Epelsiban.

Interestingly, Li et al. recently conducted a study radiologically assessing the changes in blood flow in adenomyosis after administering high doses of oxytocin [91]. They observed a significant decrease in the blood flow volume of adenomyotic lesions and recommended a continuous intravenous infusion of 0.12 U/min of oxytocin [91]. Further evidence and trials are needed to properly evaluate the role of oxytocin as a treatment of adenomyosis.

#### 3.2.6. Signal Transducer and Activator of Transcription 3 (STAT3) Inhibition

STAT3 has been proven to highly affect endometrial tissue growth [12]. Hiraoka et al. investigated the influence of STAT3 on adenomyosis in a mouse model of adenomyosis and human specimens of eutopic endometria and adenomyotic lesions. They found that mice with eutopic and ectopic endometria demonstrated positive immunoreactivity for phosphorylated STAT3 (pSTAT3), the active form of STAT3 [12]. Additionally, in humans, pSTAT3 was expressed intensely in both the eutopic endometrium and adenomyotic lesions. Hiraoka et al. concluded by suggesting that continuous STAT3 activation promoted adenomyosis development and that STAT3 inhibition could be a promising treatment strategy in patients suffering from adenomyosis [12].

#### 3.2.7. KRAS Genetically Guided Therapy

Inoue et al. applied next-generation sequencing to human adenomyosis samples, in addition to co-occurring leiomyoma and endometriosis, to evaluate the presence of somatic genomic alterations [13]. They found that KRAS mutations are more frequent in cases of adenomyosis with co-occurring endometriosis, low progesterone receptor expression, or dienogest pretreatment [13]. The authors suggested that KRAS status could be a biomarker of treatment efficacy, stating that lesions containing numerous KRAS mutations may reduce dienogest efficacy [13]. These findings could lead to genetically guided therapies and/or relapse risk assessment after uterine-sparing surgery [13]. However, these observations require further evidence to determine the validity of their hypothesis and its clinical applicability.

#### 3.2.8. Qiu’s Neiyi Recipe

Qiu’s Neiyi recipe (Qiu) is a traditional Chinese medicine that has been used for endometriosis therapy in China for decades [92]. The advantages of using traditional Chinese medicine are usually milder adverse reactions, and relatively cost-efficient prices [93]. In their study on adenomyosis in mice models, Ying et al. hypothesized that the administration of Qiu might improve the inflammation in adenomyosis through the regulation of the mitogen-activated protein kinases/extracellular signal-regulated kinases (MAPKs/ERKs) signaling pathway [94]. They demonstrated that Qiu treatment led to an improvement in symptoms by reducing myometrial infiltration, in addition to reduced levels of IL-1β, IL-6, and TNF-α in mice serum and uterine tissue [94]. Qiu alleviated the inflammatory response and uterine histological changes in mice with adenomyosis, through inhibition of the MAPK/ERK signaling pathway [94]. These results suggest that Qiu may have a role in the clinical treatment of adenomyosis.

#### 3.2.9. Micro RNA

MicroRNA therapy is a relatively new technology, with the first-ever small RNA-based therapeutic drug obtaining its FDA approval in 2018 [95]. MicroRNA therapy specifically targets and silences multiple genes, including those involved in disease development [96]. Several dysregulated microRNAs have been identified in the endometrium of adenomyosis patients [95]. This makes it reasonable to suggest the transcriptional regulation of dysregulated microRNA as a treatment for the disease [96]. However, there remains significant effort to be made to progress from in vitro studies to drug development for clinical use.

#### 3.2.10. Valproic Acid

The current evidence concerning endometriosis and adenomyosis is leaning towards them being epigenetic diseases with aberrant methylation [65,97,98,99]. Considering these findings, both diseases could be addressed by using demethylating agents and histone deacetylase inhibitors (HDACIs), such as valproic acid [100,101,102]. Valproic acid has known and favorable pharmacokinetic properties and has been used for decades to treat epilepsy and bipolar disorders [103,104].

Liu et al. evaluated the effect of Valproic acid in mice with adenomyosis, induced by neonatal tamoxifen [105]. Three weeks after treatment, they demonstrated that valproic acid significantly decreased generalized hyperalgesia [105]. Liu et al. described valproic acid as a promising therapy for the treatment of adenomyosis [105]. Additionally, they looked into the off-label use of valproic acid in patients with adenomyosis suffering from severe dysmenorrhea [106]. Two months after treatment initiation, two patients reported complete relief of dysmenorrhea, with the third patient reporting significant pain relief. At a three-month follow-up, all three patients reported complete relief of pain without the need for analgesics [106]. Ultrasound findings revealed an average reduction of one-third of the uterine volume [106]. These same findings were demonstrated in Liu et al.’s case series involving twelve patients [107]. Thus, valproic acid seems to be a promising drug in the treatment of adenomyosis.

#### 3.2.11. Levo-Tetrahydropalmatine (L-THP) and Andrographolide

L-THP and andrographolide are both active ingredients derived from Chinese medicinal herbs, used in traditional Chinese medicine [108]. L-THP is a known analgesic with a remarkable non-addictive sedative effect that has been reported to suppress uterine contraction in virgin rat models [108,109]. Additionally, L-THP was reported to significantly reduce lesion size and pain in rats with induced endometriosis [110]. Andrographolide is a potent nuclear factor kappa B (NF-kB) inhibitor used as an anti-inflammatory agent [108]. Reports have emerged stating that NF-kB p65 subunit expression is elevated in adenomyosis, suggesting that NF-kB is constitutively activated in adenomyosis [111].

Mao et al. hypothesized that both agents could reduce hyperalgesia and suppress myometrial infiltration of endometrial implants in mice with induced adenomyosis [108]. They found that treatment with L-THP and/or andrographolide suppressed myometrial infiltration, improved generalized hyperalgesia, and reduced the amplitude and irregularity of uterine contractions that contribute to dysmenorrhea in patients with adenomyosis [108]. Additionally, joint treatment with L-THP and andrographolide was the most efficacious regimen [108]. These findings suggest that L-THP and andrographolide could be promising agents for the symptomatic treatment of adenomyosis [108].

#### 3.2.12. VEGF Inhibitors

Angiogenesis plays a pivotal role in the implantation and development of ectopic endometrial lesions. The potential therapeutic usefulness of Bevacizumab (Avastin^®^), a monoclonal antibody directed against vascular endothelial growth (VEGF), in endometriosis has been speculated. Animal experiments showed beneficial effects both in the treatment and prophylaxis of endometriosis relapse [65,110,111]. Recently, we published the first report on the use of Bevacizumab in the treatment of a patient affected by severe endometriosis. A good tolerability profile and a favorable effect on pain control were recorded. Furthermore, we documented a regress in metabolic activity and increased expression of hormonal receptors in endometriosis tissue [111,112]. Overall, fighting angiogenesis with Bevacizumab seems a promising treatment for severe endometriosis. Biological and clinical effects in humans, including the safety profile, should be better evaluated.

## 4. Conclusions

Adenomyosis is a serious and debilitating disease that profoundly affects the quality of life and fertility of affected women. This review comprehensively addresses current medical treatments for adenomyosis, including NSAIDs, LNG-IUDs, progestins, and combined OCPs, as well as both GnRH agonists and antagonists. Additionally, we explore prospective and potential therapies (Table 1), such as aromatase inhibitors, selective progesterone receptor modulators, anti-platelet therapy, dopamine, oxytocin antagonists, and therapies guided by STAT3, KRAS, and MAPK, micro-RNA, valproic acid, L-THP, and andrographolide.

Available data suggest that LNG-IUDs are the most well-evaluated and efficacious treatment for adenomyosis-related symptoms when compared to other oral agents. While Dienogest, which has been studied more extensively in endometriosis, still shows favorable outcomes in adenomyosis cases, the absence of a standardized protocol remains a challenge. GnRH agonists represent a second-line option with positive outcomes, but their hypoestrogenic side effects contribute to high discontinuation rates, necessitating add-back therapy for long-term use. Promising results have also emerged for GnRH antagonists, oxytocin, and prolactin modulators, yet further studies are imperative to assess their efficacy and establish standardized protocols.

Furthermore, this review underscores the significance of continuous STAT3 activation in promoting adenomyosis and the intriguing hypothesis that KRAS mutations could potentially serve as biomarkers for treatment efficacy, opening doors to genetically guided therapies.

In recent decades, advancements in imaging studies have led to an increased number of women diagnosed with adenomyosis, heightening interest in more conservative management options. However, the scarcity of high-quality studies in this domain leaves women seeking fertility-preserving options with limited choices. As of today, hysterectomy remains the sole definitive treatment for adenomyosis, and clear guidance regarding optimal conservative management is notably lacking. Therefore, further research and efforts are necessary to provide clinicians with improved guidance for managing women with adenomyosis and to standardize their approach to this challenging condition.

## Figures and Tables

**Table 1:** Summarizing Prospective and future treatments.

Treatment	Mode of Action
1. Aromatase inhibitors	Halt the production of estrogen by blocking aromatization of androgen to estrogen. Comparative results to GnRH agonist, 60% reduced uterine volume when combined.
2. Selective Progesterone Receptor Modulators (SPRMs)	Derived from norethindrone, interact with progesterone receptors to either activate or repress gene transcription in a tissue specific manner. e.g., Ulipristal, Mifepristone
3. Antiplatelet therapy	Induces the TGF-β/Smad signaling causing fibrosis and smooth muscle metaplasia.
4. Dopamine agonist:	Inhibits prolactin. Prolactin receptors are increased in adenomyotic tissue, suggesting an association between prolactin and adenomyosis.
5. Oxytocin antagonists	Decreases myometrial peristalsis via blocking oxytocin and vasopressin V1a receptors. e.g., Atosiban, Epelsiban
6. Signal transducer and activator of transcription 3 (STAT3) inhibition:	Mice eutopic and ectopic endometria demonstrated a positive immunoreactivity for phosphorylated STAT3 (pSTAT3), the active form of STAT3. STAT3 inhibition could be a promising treatment.
7. KRAS genetically guided therapy	KRAS mutations co-occur with endometriosis, low progesterone receptor expression, or dienogest pretreatment. KRAS status could be a biomarker of treatment efficacy.
8. Qiu’s Neiyi recipe:	A traditional Chinese medicine used for endometriosis. improve the inflammation by regulation of the mitogen-activated protein kinases/extracellular signal-regulated kinases (MAPKs/ERKs) pathways.
9. Micro RNA:	MicroRNA therapy specifically targets and silences multiple genes. transcriptional regulation of dysregulated microRNA in adenomyosis is proposed
10. Valproic Acid:	Demethylating agent and histone deacetylase inhibitor (HDACIs). Improves hyperalgesia, dysmenorrhea and reduce uterine volume.
11. Levo-tetrahydropalmatine (L-THP) and andrographolide:	Chinese medicinal herbs. L-THP is a known analgesic and Andrographolide potent nuclear factor kappa B (NF-kB) inhibitor is an anti-inflammatory agent.
12. Vascular endothelial growth (VEGF) inhibitors:	Bevacizumab (Avastin®) is a monoclonal antibody directed against VEGF in endometriosis with potential usefulness in adenomyosis.

## Data Availability

All data generated or analyzed during this study are included in this published article.

## References

[1] Agostinho L., Cruz R., Osório F., Alves J., Setúbal A., Guerra A. (2017). MRI for adenomyosis: A pictorial review. Insights Into Imaging.

[2] Akira S., Mine K., Kuwabara Y., Takeshita T. (2008). Efficacy of long-term, low-dose gonadotropin-releasing hormone agonist therapy (draw-back therapy) for adenomyosis. Med. Sci. Monit..

[3] Andersson J.K., Khan Z., Weaver A.L., Vaughan L.E., Gemzell-Danielsson K., Stewart E.A. (2019). Vaginal bromocriptine improves pain, menstrual bleeding and quality of life in women with adenomyosis: A pilot study. Acta Obstet. Gynecol. Scand..

[4] Andersson J.K., Mucelli R.P., Epstein E., Stewart E.A., Gemzell-Danielsson K. (2020). Vaginal bromocriptine for treatment of adenomyosis: Impact on magnetic resonance imaging and transvaginal ultrasound. Eur. J. Obstet. Gynecol. Reprod. Biol..

[5] Ascher S.M., Arnold L.L., Patt R.H., Schruefer J.J., Bagley A.S., Semelka R.C., Zeman R.K., Simon J.A. (1994). Adenomyosis: Prospective comparison of MR imaging and transvaginal sonography. Radiology.

[6] Atri M., Reinhold C., Mehio A.R., Chapman W.B., Bret P.M. (2000). Adenomyosis: US features with histologic correlation in an in-vitro study. Radiology.

[7] Badawy A.M., Elnashar A.M., Mosbah A.A. (2012). Aromatase inhibitors or gonadotropin-releasing hormone agonists for the management of uterine adenomyosis: A randomized controlled trial. Acta Obstet. Gynecol. Scand..

[8] Bazot M., Cortez A., Darai E., Rouger J., Chopier J., Antoine J.-M., Uzan S. (2001). Ultrasonography compared with magnetic resonance imaging for the diagnosis of adenomyosis: Correlation with histopathology. Hum. Reprod..

[9] Beatty M.N., Blumenthal P.D. (2009). The levonorgestrel-releasing intrauterine system: Safety, efficacy, and patient acceptability. Ther. Clin. Risk Manag..

[10] Bromley B., Shipp T.D., Benacerraf B. (2000). Adenomyosis: Sonographic findings and diagnostic accuracy. J. Ultrasound. Med..

[11] Bulun S.E., Yildiz S., Adli M., Chakravarti D., Parker J.B., Milad M., Yang L., Chaudhari A., Tsai S., Wei J.J. (2023). Endometriosis and adenomyosis: Shared pathophysiology. Fertil. Steril..

[12] Burney R.O., Giudice L.C. (2012). Pathogenesis and pathophysiology of endometriosis. Fertil. Steril..

[13] Calderon L., Netter A., Grob-Vaillant A., Mancini J., Siles P., Vidal V., Agostini A. (2021). Progression of adenomyosis magnetic resonance imaging features under ulipristal acetate for symptomatic fibroids. Reprod. BioMed. Online.

[14] Capmas P., Brun J.-L., Legendre G., Koskas M., Merviel P., Fernandez H. (2021). Ulipristal acetate use in adenomyosis: A randomized controlled trial. J. Gynecol. Obstet. Hum. Reprod..

[15] Chapron C., Tosti C., Marcellin L., Bourdon M., Lafay-Pillet M.-C., Millischer A.-E., Streuli I., Borghese B., Petraglia F., Santulli P. (2017). Relationship between the magnetic resonance imaging appearance of adenomyosis and endometriosis phenotypes. Hum. Reprod..

[16] Che X., Wang J., He J., Guo X., Li T., Zhang X. (2020). The new application of mifepristone in the relief of adenomyosis-caused dysmenorrhea. Int. J. Med. Sci..

[17] Chen C., Wu D., Guo Z., Xie Q., Reinhart G.J., Madan A., Wen J., Chen T., Huang C.Q., Chen M. (2008). Discovery of Sodium R-(+)-4-{2-[5-(2-Fluoro-3-methoxyphenyl)-3-(2-fluoro-6-[trifluoromethyl] benzyl)-4-methyl-2, 6-dioxo-3, 6-dihydro-2 H-pyrimidin-1-yl]-1-phenylethylamino} butyrate (Elagolix), a Potent and Orally Available Nonpeptide Antagonist of the Human Gonadotropin-Releasing Hormone Receptor. J. Med. Chem..

[18] Chu H., Jin G., Friedman E., Zhen X. (2007). Recent development in studies of tetrahydroprotoberberines: Mechanism in antinociception and drug addiction. Cell. Mol. Neurobiol..

[19] Conway F., Morosetti G., Camilli S., Martire F.G., Sorrenti G., Piccione E., Zupi E., Exacoustos C. (2019). Ulipristal acetate therapy increases ultrasound features of adenomyosis: A good treatment given in an erroneous diagnosis of uterine fibroids. Gynecol. Endocrinol..

[20] Dessouky R., Gamil S.A., Nada M.G., Mousa R., Libda Y. (2019). Management of uterine adenomyosis: Current trends and uterine artery embolization as a potential alternative to hysterectomy. Insights Into Imaging.

[21] Di Donato N., Montanari G., Benfenati A., Leonardi D., Bertoldo V., Monti G., Raimondo D., Seracchioli R. (2014). Prevalence of adenomyosis in women undergoing surgery for endometriosis. Eur. J. Obstet. Gynecol. Reprod. Biol..

[22] Diamond Michael P., Carr B., Dmowski W.P., Koltun W., O’Brien C., Jiang P., Burke J., Jimenez R., Garner E., Chwalisz K. (2014). Elagolix treatment for endometriosis-associated pain: Results from a phase 2, randomized, double-blind, placebo-controlled study. Reprod. Sci..

[23] Donnez J., Dolmans M.-M. (2016). Uterine fibroid management: From the present to the future. Hum. Reprod. Update.

[24] Donnez J., Donnez O., Dolmans M.-M. (2016). Safety of treatment of uterine fibroids with the selective progesterone receptor modulator, ulipristal acetate. Expert Opin. Drug Saf..

[25] Donnez J., Tatarchuk T.F., Bouchard P., Puscasiu L., Zakharenko N.F., Ivanova T., Ugocsai G., Mara M., Jilla M.P., Bestel E. (2012). Ulipristal Acetate versus Placebo for Fibroid Treatment before Surgery. N. Engl. J. Med..

[26] Donnez J., Tomaszewski J., Vázquez F., Bouchard P., Lemieszczuk B., Baró F., Nouri K., Selvaggi L., Sodowski K., Bestel E. (2012). Ulipristal Acetate versus Leuprolide Acetate for Uterine Fibroids. N. Engl. J. Med..

[27] Donnez J., Vázquez F., Tomaszewski J., Nouri K., Bouchard P., Fauser B.C., Barlow D.H., Palacios S., Donnez O., Bestel E. (2014). Long-term treatment of uterine fibroids with ulipristal acetate. Fertil. Steril..

[28] Donnez O., Donnez J. (2020). Gonadotropin-releasing hormone antagonist (linzagolix): A new therapy for uterine adenomyosis. Fertil. Steril..

[29] Dueholm M., Lundorf E., Hansen E.S., Sørensen J.S., Ledertoug S., Olesen F. (2001). Magnetic resonance imaging and transvaginal ultrasonography for the diagnosis of adenomyosis. Fertil. Steril..

[30] Farquhar C., Brosens I. (2006). Medical and surgical management of adenomyosis. Best Pract. Res. Clin. Obstet. Gynaecol..

[31] Fauser B.C.J.M., Donnez J., Bouchard P., Barlow D.H., Vázquez F., Arriagada P., Skouby S.O., Palacios S., Tomaszewski J., Lemieszczuk B. (2017). Safety after extended repeated use of ulipristal acetate for uterine fibroids. PLoS ONE.

[32] García-Solares J., Donnez J., Donnez O., Dolmans M.-M. (2018). Pathogenesis of uterine adenomyosis: Invagination or metaplasia?. Fertil. Steril..

[33] Gottlicher M., Minucci S., Zhu P., Kramer O.H., Schimpf A., Giavara S., Sleeman J.P., Coco F.L., Nervi C., Pelicci P.G. (2002). The teratogen valproic acid defines a novel class of histone deacetylase inhibitors inducing differentiation of embryonic, transformed and tumorigenic cells. Naunyn-Schmiedeberg’s Archives of Pharmacology.

[34] Greaves P., White I.N.H. (2006). Experimental adenomyosis. Best Pract. Res. Clin. Obstet. Gynaecol..

[35] Gruber T.M., Mechsner S. (2021). Pathogenesis of Endometriosis: The Origin of Pain and Subfertility. Cells.

[36] Güzel A.I., Akselim B., Erkılınç S., Kokanalı K., Tokmak A., Dolmuş B., Doğanay M. (2015). Risk factors for adenomyosis, leiomyoma and concurrent adenomyosis and leiomyoma. J. Obstet. Gynaecol. Res..

[37] Hauksson A., Ekström P., Juchnicka E., Laudanski T., Åkerlund M., Åkerlund D.M. (1989). The influence of a combined oral contraceptive on uterine activity and reactivity to agonists in primary dysmenorrhea. Acta Obstet. Gynecol. Scand..

[38] Herndon C.N., Aghajanova L., Balayan S., Erikson D., Bs F.B., Goldfien G., Vo K.C., Hawkins S., Giudice L.C. (2016). Global Transcriptome Abnormalities of the Eutopic Endometrium from Women With Adenomyosis. Reprod. Sci..

[39] Hiraoka T., Hirota Y., Fukui Y., Gebril M., Kaku T., Aikawa S., Hirata T., Akaeda S., Matsuo M., Haraguchi H. (2020). Differential roles of uterine epithelial and stromal STAT3 coordinate uterine receptivity and embryo attachment. Sci. Rep..

[40] Igarashi M. (2002). Further studies on danazol-loaded IUD in uterine adenomyosis. Fertil. Steril..

[41] Igarashi M. (1990). A New Therapy for Pelvic Endometriosis and Uterine Adenomyosis: Local Effect of Vaginal and Intrauterine Danazol Application. Asia-Ocean. J. Obstet. Gynaecol..

[42] Igarashi M., Abe Y., Fukuda M., Ando A., Miyasaka M., Yoshida M. (2000). Novel conservative medical therapy for uterine adenomyosis with a danazol-loaded intrauterine device. Fertil. Steril..

[43] Imai A., Matsunami K., Takagi H., Ichigo S. (2014). Levonorgestrel-releasing intrauterine device used for dysmenorrhea: Five-year literature review. Clin. Exp. Obstet. Gynecol..

[44] Inoue S., Hirota Y., Ueno T., Fukui Y., Yoshida E., Hayashi T., Kojima S., Takeyama R., Hashimoto T., Kiyono T. (2019). Uterine adenomyosis is an oligoclonal disorder associated with KRAS mutations. Nat. Commun..

[45] Ishihara H., Kitawaki J., Kado N., Koshiba H., Fushiki S., Honjo H. (2003). Gonadotropin-releasing hormone agonist and danazol normalize aromatase cytochrome P450 expression in eutopic endometrium from women with endometriosis, adenomyosis, or leiomyomas. Fertil. Steril..

[46] Johannessen C.U. (2000). Mechanisms of action of valproate: A commentatory. Neurochem. Int..

[47] Kavoussi S.K., Esqueda A.S., Jukes L.M. (2020). Elagolix to medically treat a uterine adenomyoma: A case report. Eur. J. Obstet. Gynecol. Reprod. Biol..

[48] Kepkep K., Tuncay Y.A., Göynümer G., Tutal E. (2007). Transvaginal sonography in the diagnosis of adenomyosis: Which findings are most accurate?. Ultrasound Obstet. Gynecol..

[49] Khan K.N., Kitajima M., Hiraki K., Fujishita A., Sekine I., Ishimaru T., Masuzaki H. (2009). Changes in tissue inflammation, angiogenesis and apoptosis in endometriosis, adenomyosis and uterine myoma after GnRH agonist therapy. Hum. Reprod..

[50] Kimura F., Takahashi K., Takebayashi K., Fujiwara M., Kita N., Noda Y., Harada N. (2007). Concomitant treatment of severe uterine adenomyosis in a premenopausal woman with an aromatase inhibitor and a gonadotropin-releasing hormone agonist. Fertil. Steril..

[51] Kitawaki J. (2006). Adenomyosis: The pathophysiology of an oestrogen-dependent disease. Best Pract. Res. Clin. Obstet. Gynaecol..

[52] Koninckx P.R., Ussia A., Adamyan L., Tahlak M., Keckstein J., Wattiez A., Martin D.C. (2020). The epidemiology of endometriosis is poorly known as the pathophysiology and diagnosis are unclear. Best Pract. Res. Clin. Obstet. Gynaecol..

[53] Lethaby A., Duckitt K., Farquhar C. (2019). Non-steroidal anti-inflammatory drugs for heavy menstrual bleeding. Cochrane Database Syst Rev..

[54] Levgur M. (2007). Diagnosis of adenomyosis: A review. J. Reprod. Med..

[55] Li J., Chen J., Wang Y., Hu L., Zhang R., Chen W. (2022). Doppler Imaging Assessment of Changes of Blood Flow in Adenomyosis After Higher-Dose Oxytocin: A Randomized Controlled Trial. J. Ultrasound Med..

[56] Liu X., Shen M., Qi Q., Zhang H., Guo S.-W. (2016). Corroborating evidence for platelet-induced epithelial-mesenchymal transition and fibroblast-to-myofibroblast transdifferentiation in the development of adenomyosis. Hum. Reprod..

[57] Liu X., Guo S.-W. (2008). A pilot study on the off-label use of valproic acid to treat adenomyosis. Fertil. Steril..

[58] Liu X., Guo S.-W. (2011). Valproic acid alleviates generalized hyperalgesia in mice with induced adenomyosis. J. Obstet. Gynaecol. Res..

[59] Loo M.H., Egan D., Vaughan E.D., Marion D., Felsen D., Weisman S. (1987). The Effect of the Thromboxane A2 Synthesis Inhibitor Oky-046 on Renal Function in Rabbits Following Release of Unilateral Ureteral Obstruction. J. Urol..

[60] Lu A.-P., Jia H.-W., Xiao C., Lu Q.-P. (2004). Theory of traditional Chinese medicine and therapeutic method of diseases. World J. Gastroenterol..

[61] Mahar K.M., Enslin M.B., Gress A., Amrine-Madsen H., Cooper M. (2017). Single- and Multiple-Day Dosing Studies to Investigate High-Dose Pharmacokinetics of Epelsiban and Its Metabolite, GSK2395448, in Healthy Female Volunteers. Clin. Pharmacol. Drug Dev..

[62] Mansouri R., Santos X.M., Bercaw-Pratt J.L., Dietrich J.E. (2015). Regression of Adenomyosis on Magnetic Resonance Imaging after a Course of Hormonal Suppression in Adolescents: A Case Series. J. Pediatr. Adolesc. Gynecol..

[63] Mao X., Wang Y., Carter A.V., Zhen X., Guo S.-W. (2011). The Retardation of Myometrial Infiltration, Reduction of Uterine Contractility, and Alleviation of Generalized Hyperalgesia in Mice with Induced Adenomyosis by Levo-Tetrahydropalmatine (l-THP) and Andrographolide. Reprod. Sci..

[64] Marjoribanks J., Ayeleke R.O., Farquhar C., Proctor M. (2015). Nonsteroidal anti-inflammatory drugs for dysmenorrhoea. Cochrane Database Syst. Rev..

[65] Nie J., Lu Y., Liu X., Guo S.-W. (2009). Immunoreactivity of progesterone receptor isoform B, nuclear factor κB, and IκBα in adenomyosis. Fertil. Steril..

[66] Jichan N., Liu X., Guo S.-W. (2010). Immunoreactivity of oxytocin receptor and transient receptor potential vanilloid type 1 and its correlation with dysmenorrhea in adenomyosis. Am. J. Obstet. Gynecol..

[67] Asher O., Weber-Schöndorfer C., Schaefer C., Peters P., Miller R.K. (2015). 2.15—Hormones. Drugs during Pregnancy and Lactation.

[68] Osuga Y., Fujimoto-Okabe H., Hagino A. (2017). Evaluation of the efficacy and safety of dienogest in the treatment of painful symptoms in patients with adenomyosis: A randomized, double-blind, multicenter, placebo-controlled study. Fertil. Steril..

[69] Osuga Y., Hayashi K., Kanda S. (2020). Long-term use of dienogest for the treatment of primary and secondary dysmenorrhea. J. Obstet. Gynaecol. Res..

[70] Osuga Y., Watanabe M., Hagino A. (2017). Long-term use of dienogest in the treatment of painful symptoms in adenomyosis. J. Obstet. Gynaecol. Res..

[71] Ozdegirmenci O., Kayikcioglu F., Akgul M.A., Kaplan M., Karcaaltincaba M., Haberal A., Akyol M. (2011). Comparison of levonorgestrel intrauterine system versus hysterectomy on efficacy and quality of life in patients with adenomyosis. Fertil. Steril..

[72] Park C.W., Choi M.H., Yang K.M., Song I.O. (2016). Pregnancy rate in women with adenomyosis undergoing fresh or frozen embryo transfer cycles following gonadotropin-releasing hormone agonist treatment. Clin. Exp. Reprod. Med..

[73] Phiel C.J., Zhang F., Huang E.Y., Guenther M.G., Lazar M.A., Klein P.S. (2001). Histone deacetylase is a direct target of valproic acid, a potent anticonvulsant, mood stabilizer, and teratogen. J. Biol. Chem..

[74] Pontis A., D’Alterio M.N., Pirarba S., de Angelis C., Tinelli R., Angioni S. (2016). Adenomyosis: A systematic review of medical treatment. Gynecol. Endocrinol..

[75] Qin X., Sun W., Wang C., Li M., Zhao X., Li C., Zhang H. (2021). Mifepristone inhibited the expression of B7-H2, B7-H3, B7-H4 and PD-L2 in adenomyosis. Reprod. Biol. Endocrinol..

[76] Reinhold C., Atri M., Mehio A., Zakarian R., Aldis A.E., Bret P.M. (1995). Diffuse uterine adenomyosis: Morphologic criteria and diagnostic accuracy of endovaginal sonography. Radiology.

[77] Reinhold C., McCarthy S., Bret P.M., Mehio A., Atri M., Zakarian R., Glaude Y., Liang L., Seymour R.J. (1996). Diffuse adenomyosis: Comparison of endovaginal US and MR imaging with histopathologic correlation. Radiology.

[78] Reinhold C., Tafazoli F., Mehio A., Wang L., Atri M., Siegelman E.S., Rohoman L. (1999). Uterine adenomyosis: Endovaginal US and MR imaging features with histopathologic correlation. Radiographics.

[79] Shaaban O.M., Ali M.K., Sabra A.M.A., El Aal D.E.M.A. (2015). Levonorgestrel-releasing intrauterine system versus a low-dose combined oral contraceptive for treatment of adenomyotic uteri: A randomized clinical trial. Contraception.

[80] Sharara F.I., Kheil M.H., Feki A., Rahman S., Klebanoff J.S., Ayoubi J.M., Moawad G.N. (2021). Current and Prospective Treatment of Adenomyosis. J. Clin. Med..

[81] Shawki O., Igarashi M. (2002). Danazol loaded intrauterine device (IUD) for management of uterine adenomyosis: A novel approach. Fertil. Steril..

[82] Shen M., Liu X., Zhang H., Guo S.-W. (2016). Transforming growth factor β1 signaling coincides with epithelial-mesenchymal transition and fibroblast-to-myofibroblast transdifferentiation in the development of adenomyosis in mice. Hum. Reprod..

[83] Sheng J., Zhang W.Y., Zhang J.P., Lu D. (2009). The LNG-IUS study on adenomyosis: A 3-year follow-up study on the efficacy and side effects of the use of levonorgestrel intrauterine system for the treatment of dysmenorrhea associated with adenomyosis. Contraception.

[84] Spitz I.M., Grunberg S.M., Chabbert-Buffet N., Lindenberg T., Gelber H., Sitruk-Ware R. (2005). Management of patients receiving long-term treatment with mifepristone. Fertil. Steril..

[85] Stratopoulou C.A., Donnez J., Dolmans M.-M. (2021). Origin and Pathogenic Mechanisms of Uterine Adenomyosis: What Is Known So Far. Reprod. Sci..

[86] Stratopoulou C.A., Donnez J., Dolmans M.-M. (2021). Conservative Management of Uterine Adenomyosis: Medical vs. Surgical Approach. J. Clin. Med..

[87] Streuli I., Dubuisson J., Santulli P., de Ziegler D., Batteux F., Chapron C. (2014). An update on the pharmacological management of adenomyosis. Expert. Opin. Pharmacother..

[88] Struthers R.S., Nicholls A.J., Grundy J., Chen T., Jimenez R., Yen S.S.C., Bozigian H.P. (2009). Suppression of gonadotropins and estradiol in premenopausal women by oral administration of the nonpeptide gonadotropin-releasing hormone antagonist elagolix. J. Clin. Endocrinol. Metab..

[89] Sun X., Bartos A., Whitsett J.A., Dey S.K. (2013). Uterine deletion of Gp130 or Stat3 shows implantation failure with increased estrogenic responses. Mol. Endocrinol..

[90] Szyf M. (2005). Therapeutic implications of DNA methylation. Future Med..

[91] Taran F.A., Stewart E.A., Brucker S. (2013). Adenomyosis: Epidemiology, Risk Factors, Clinical Phenotype and Surgical and Interventional Alternatives to Hysterectomy. Geburtshilfe Frauenheilkd..

[92] Tsui K.-H., Lee W.-L., Chen C.-Y., Sheu B.-C., Yen M.-S., Chang T.-C., Wang P.-H. (2014). Medical treatment for adenomyosis and/or adenomyoma. Taiwan. J. Obstet. Gynecol..

[93] Tunnicliff G. (1999). Actions of sodium valproate on the central nervous system. J. Physiol. Pharmacol..

[94] Vannuccini S., Luisi S., Tosti C., Sorbi F., Petraglia F. (2018). Role of medical therapy in the management of uterine adenomyosis. Fertil. Steril..

[95] Vannuccini S., Petraglia F. (2019). Recent advances in understanding and managing adenomyosis. F1000Research.

[96] Vannuccini S., Tosti C., Carmona F., Huang S.J., Chapron C., Guo S.-W., Petraglia F. (2017). Pathogenesis of adenomyosis: An update on molecular mechanisms. Reprod. Biomed. Online.

[97] Wang Y., Jiang X., Wang S. (2014). The influence of mifepristone to caspase 3 expression in adenomyosis. Clin. Exp. Obs. Gynecol..

[98] Wong C.L., Farquhar C., Roberts H., Proctor M. (2009). Oral contraceptive pill for primary dysmenorrhoea. Cochrane Database Syst. Rev..

[99] Wu Y., Halverson G., Basir Z., Strawn E., Yan P., Guo S.-W. (2005). Aberrant methylation at HOXA10 may be responsible for its aberrant expression in the endometrium of patients with endometriosis. Am. J. Obstet. Gynecol..

[100] Wu Y., Strawn E., Basir Z., Halverson G., Guo S.-W. (2006). Promoter hypermethylation of progesterone receptor isoform B (PR-B) in endometriosis. Epigenetics.

[101] Wu Y., Strawn E., Basir Z., Halverson G., Guo S.W. (2007). Aberrant expression of deoxyribonucleic acid methyltransferases DNMT1, DNMT3A, and DNMT3B in women with endometriosis. Fertil. Steril..

[102] Liu X., Yuan L., Guo S.-W. (2010). Valproic Acid as a Therapy for Adenomyosis: A Comparative Case Series. Reprod. Sci..

[103] Ying P., Li H., Jiang Y., Yao Z., Lu S., Yang H., Zhu Y. (2021). Qiu’s Neiyi Recipe Regulates the Inflammatory Action of Adenomyosis in Mice via the MAPK Signaling Pathway. Evid. Based Complement. Altern. Med. Ecam.

[104] Younes G., Tulandi T. (2017). Effects of adenomyosis on in vitro fertilization treatment outcomes: A meta-analysis. Fertil. Steril..

[105] Yu O., Schulze-Rath R., Grafton J., Hansen K., Scholes D., Reed S.D. (2020). Adenomyosis incidence, prevalence and treatment: United States population-based study 2006-2015. Am. J. Obs. Gynecol..

[106] Zhai J., Vannuccini S., Petraglia F., Giudice L.C. (2020). Adenomyosis: Mechanisms and pathogenesis. Semin. Reprod. Med..

[107] Zhao T., Liu X., Zhen X., Guo S.-W. (2011). Levo-tetrahydropalmatine retards the growth of ectopic endometrial implants and alleviates generalized hyperalgesia in experimentally induced endometriosis in rats. Reprod. Sci..

[108] Zhu B., Chen Y., Shen X., Liu X., Guo S.-W. (2016). Anti-platelet therapy holds promises in treating adenomyosis: Experimental evidence. Reprod. Biol. Endocrinol. RBE.

[109] Zhu Y.P., Wu Y.P. (2013). Effect of Neiyi prescription of QIU on expressions of matrix metalloproteinase-2 and tissue inhibitor of metalloproteinase-2 in rats with endometriosis. Chin. Arch. Tradit. Chin. Med..

[110] Łupicka M., Socha B., Szczepańska A., Korzekwa A. (2017). Prolactin role in the bovine uterus during adenomyosis. Domest. Anim. Endocrinol..

[111] Zani A.C.T., Valerio F.P., Meola J., da Silva A.R., Nogueira A.A., Candido-dos-Reis F.J., Poli-Neto O.B., Poli-Neto J.C. (2020). Impact of Bevacizumab on Experimentally Induced Endometriotic Lesions: Angiogenesis, Invasion, Apoptosis, and Cell Proliferation. Reprod Sci..

[112] Bouquet de Joliniere J., Fruscalzo A., Khomsi F., Stochino Loi E., Cherbanyk F., Ayoubi J.M., Feki A. (2021). Antiangiogenic Therapy as a New Strategy in the Treatment of Endometriosis? The First Case Report. Front Surg..

